# Laryngopharyngeal pH Monitoring in Patients With Idiopathic Pulmonary Fibrosis

**DOI:** 10.3389/fphar.2021.724286

**Published:** 2021-08-12

**Authors:** Yiliang Su, Li Shen, Fen Zhang, Xing Jiang, Xiaofeng Jin, Yuan Zhang, Yang Hu, Ying Zhou, Qiuhong Li, Huiping Li

**Affiliations:** Department of Respiratory and Critical Care Medicine, Shanghai Pulmonary Hospital, School of Medicine, Tongji University, Shanghai, China

**Keywords:** interstitial lung diseases, idiopathic pulmonary fibrosis, laryngopharyngeal reflux, ryan index, cough

## Abstract

**Background:** Patients with idiopathic pulmonary fibrosis (IPF) often have irritating persistent dry cough. Possible correlations between dry cough and laryngopharyngeal reflux (LPR) remain unclear.

**Methods:** 44 patients with IPF and 30 healthy individuals underwent 24 h laryngopharyngeal pH monitoring. Ryan index score was calculated. Patients’ demographic and clinical data were collected.

**Results:** 44 patients with IPF and 30 healthy individuals were included. The proportions of men and smokers were significantly higher in IPF group than control group (All *p* < 0.01). The average laryngopharyngeal pH value for 24 h was similar in the IPF (7.11 ± 0.08) group and control group (7.09 ± 0.06). According to the percentage duration of pH < 6.5, pH6.5–7.5, and pH > 7.5 in the overall measure duration, the patients were classified into three pH groups. In entire pH monitoring duration, the proportion of pH > 7.5 group in IPF patients was higher than control group; at upright position, the proportion of pH > 7.5 group in IPF patients was higher than control group; at supine position, the proportion of pH < 6.5 group in IPF patients was higher than control group (All *p* < 0.01). Seven patients had Ryan index score>9.41 at upright position. All patients had Ryan index score<6.79 at supine position. Four patients showed significantly higher and one patient had significantly lower average pH at coughing than the overall average pH (All *p* < 0.05).

**Conclusions:** Patients with IPF may have LPR. Basic and acidic LPR may likely occur at upright and supine position, respectively. Ryan index may not accurately reflect LPR in patients with IPF.

## Introduction

Interstitial lung diseases (ILDs) refer to a group of lung diseases characterized in alveolar inflammation and interstitial fibrotic lesions (Travis et al., 2013). The etiology of ILDs is complex and diverse. Idiopathic pulmonary fibrosis (IPF) and connective tissue disease-related interstitial lung disease (CTD-ILD) are the most common types of ILD (Hu et al., 2016). The main respiratory symptoms of IPF include irritating cough and breathing difficulty.

Gastroesophageal reflux disease (GERD) is caused by the reflux of gastric acid and other stomach contents into the esophagus, and one of its typical clinical symptoms is cough (Cohen et al., 2002; Hom and Vaezi, 2013; Lai et al., 2013). GERD has been recognized as a common cause for chronic cough (Lai et al., 2013). International diagnosis and treatment guidelines for IPF suggest an association between GERD and IPF onset and thus recommend anti-acid treatments for patients with IPF (Raghu et al., 2006; Lee et al., 2011; Raghu et al., 2011; Raghu et al., 2015). However, a previous report has shown that anti-acid treatments do not reduce the all-cause mortality and the rate of hospitalization of patients with IPF (Lee et al., 2013). Moreover, Raghu and colleagues have found that approximately half of patients with IPF did not present gastroesophageal reflux (GER) symptoms, such as heartburn and sour regurgitation (Raghu et al., 2011). Extraesophageal reflux (EER) or laryngopharyngeal reflux (LPR) is the reflux of stomach contents into the throat and larynx, and patients with LPR usually do not present the classic symptoms of GERD (Hom and Vaezi, 2013). An accurate diagnosis of LPR remains challenging in clinical practice.

LPR is the reflux of stomach contents into the area above the upper esophageal sphincter, such as the nasal cavity, mouth, throat, trachea, and lung (Koufman, 2002). Patients with LPR disease (LPRD) often presents cough, laryngopharyngeal discomfort, and breathing difficulty (Cohen et al., 2002; Koufman, 2002; Ayazi et al., 2009). Similar to GERD, LPRD can also cause chronic cough. Gaseous refluxate in the airway reduces the pH of the upper airway to <6.5, which activates the pepsin that is from the refluxate and deposited on the airway mucosal epithelial cells and consequently induces nonspecific inflammation in the airway mucosa (Cohen et al., 2002; Koufman, 2002). Previous studies have demonstrated that 32–84% of patients with bronchial asthma had GERD and 50% of the patients with GERD did not present any obvious reflux symptoms such as heartburn and sour regurgitation (Harding et al., 2000; Mastronarde et al., 2009). GERD can exacerbate asthma. Antacids and gastric pro-motility drugs can alleviate asthma symptoms in patients with GERD (Sharma et al., 2007; DiMango et al., 2009; Liang et al., 2013). The association between LPR and IPF remains unclear, and monitoring upper airway pH in patients with IPF is essential to understand such association and may shed light on therapeutic strategies for LPR in patients with IPF.

## Materials and Methods

### Study Design

This single-center prospective study was conducted in Shanghai Pulmonary Hospital of Tongji University in Shanghai China. A total of 44 patients with IPF treated in Shanghai Pulmonary Hospital from November 2016 to September 2017 were included in this study. The study protocol has been approved by the Ethics Committee of Shanghai Pulmonary Hospital (Approval No: K16-296). The study was registered on the Chinese Clinical Trial Registry (http://www.chictr.org.cn/abouten.aspx, ChiCTR-ODC-16009478). Written informed consent was obtained from all the study participants prior to inclusion in the study.

### Study Participants

Patients with a confirmed diagnosis of IPF were enrolled. Patients with the following clinical characteristics were excluded: 1) had a malignancy or a history of malignancy; 2) were using glucocorticoid and/or immunosuppressants (including azathioprine, cyclophosphamide, mycophenolate mofetil, and cyclosporin A); 3) had serious systemic diseases and organ dysfunction; 4) refuse to do laryngophary reflux 24 h monitor; 5) had incomplete clinical data. A total of 30 age-matched healthy individuals, who had routine physical examination in Shanghai Pulmonary Hospital, were included as controls.

### Disease Diagnostic Criteria

IPF was diagnosed according to the 2013 American Thoracic Society/European Respiratory Society statement (Travis et al., 2013).

### Twenty Four-Hour Laryngopharyngeal pH Monitoring

The Restech^®^ pH sensor (Respiratory Technology Corp., San Diego, CA) was first calibrated using two standard buffer solutions at pH 7 and pH 4, respectively. The nasal passage was topically anesthetized using Q-tips soaked with 2% lidocaine. The sensor was inserted into the nasal cavity and moved toward the throat until the flashing LED of the sensor was visible in the back of the throat, and then the sensor was positioned so that the flashing light was 5–10 mm below the uvula. The 5 mm-long LED light serves as a guide for the placement of the pH sensor. The catheter was first secured as close to the nares as possible on the face using a Tegaderm™ and then passed over the ear and secured on the neck using another Tegaderm™. The transmitter at the end of the catheter was either taped to the skin or attached to the study participant’s clothing using a clip-on case. The data recorder was attached to the study participant’s belt. The study participants were prohibited from taking a shower or bath during the recording period and were required to keep a diary to record meal periods and the time staying at supine and upright positions. The meal periods were excluded from data analyses. The data collected by the Restech® recorder and the information from the dairy were analyzed (Ayazi et al., 2009; Sun et al., 2009).

### Ryan Index Calculation

Based on the data collected from the Dx-pH 24 h laryngopharyngeal pH monitoring, a LPR episode was defined as a pharyngeal pH < 5.5 at upright position and/or <5.0 at supine position. The number of LPR episodes, the longest duration of a LPR episode, and the proportion of total duration of LPR episodes in the total recording duration (episodes % time) were calculated, and the differences in these parameters between study participants and healthy population were determined according to a previous description (Cohen et al., 2002). The data analysis software of the Restech® recorder calculates patients’ Ryan index based on the number of LPR episodes, duration of the longest LPR episode, and LPR episodes % time (Cohen et al., 2002).

### Blood Tests and Arterial Blood Gas Test

Blood tests were performed to evaluate liver and kidney function, C-reactive protein (CRP) levels, and erythrocyte sedimentation rate (ESR). Partial pressure of oxygen (PaO_2_), partial pressure of carbon dioxide (PaCO_2_), and oxygen saturation (SaO_2_) were also measured.

### Chest High-Resolution Computed Tomography Score

The scoring criteria for chest HRCT followed a previous description and are described in Table 1 (Su et al., 2017). One radiologist and two pulmonologists scored chest HRCT results separately, and the average score was used for data analyses.

**TABLE 1:** Chest HRCT scoring criteria.

Score	Lesions and lung segments
Severity score	Parenchymal abnormalities
1	Ground glass opacities
2	Irregularities in the pleural margins
3	Septal/Subpleural lines
4	Honeycombing
5	Subpleural cysts
Extent score	Number of lung segments
1	1–3
2	4–9
3	>9

HRCT score = severity score + extent score (0–30). HRCT: high resolution computed tomography.

### Pulmonary Function Test

Force vital capacity (FVC), FVC expressed as a percentage of predicted (FVC % pred), and carbon monoxide diffusing capacity of the lung expressed as a percentage of predicted (D_L_CO % pred) were determined to assess study participants’ pulmonary function.

### Cough Symptom Score

Cough symptom score (CSS) was determined according to a previous description and the criteria are displayed in Table 2 (Chung, 2006). Two pulmonologists independently evaluated daytime CSS, nighttime CSS, and total CSS. The average scores from the two pulmonologists were used for data analysis.

**TABLE 2:** Cough symptom scoring criteria.

Score	Daytime cough symptom score	Night-time cough symptom score
0	no cough during the day	no cough during the night
1	cough for infrequent short periods	short cough when falling asleep/infrequent nocturnal cough
2	frequent coughing, which did interfere with usual daytime activities	mildly interfere with nocturnal sleep due to coughs
3	frequent coughing, which did severely interfere with usual daytime activities	severely interfere with nocturnal sleep due to coughs

Total cough symptom score = Daytime cough symptom score + Night-time cough symptom score.

### Statistical Analysis

The statistical analysis software SPSS 16.0 was used. Measurement variables are presented as mean ± standard deviation (SD). Inter-group comparison was examined by independent *t*-test. One sample *t*-test was used to compare the average pH at coughing versus the overall average pH. *p* < 0.05 was considered statistically significant. The constituent ratios of different pH range (pH < 6.5, pH 6.5–7.5, and pH > 7.5) were analysed by chi-square test.

## Results

### General Clinical Data

**FIGURE 1:**
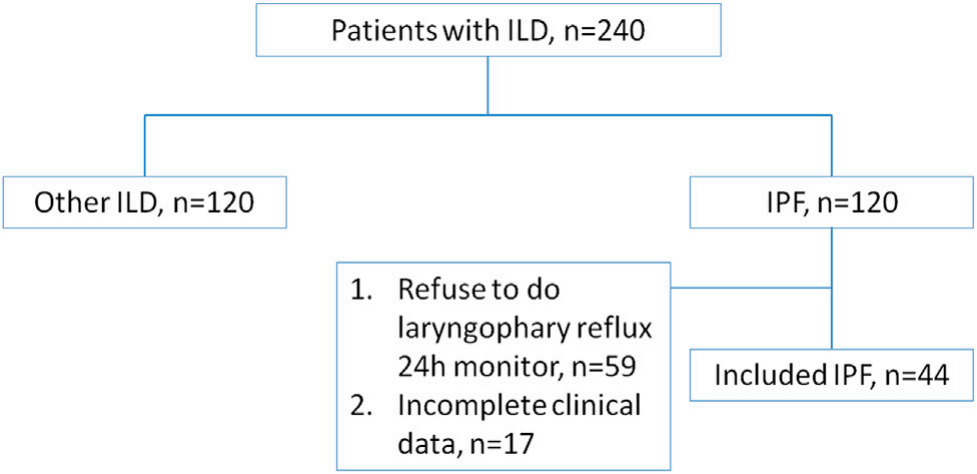
Patient flow chart.

The patient flowchart is displayed in Figure 1. A total of 44 patients with IPF were included in the study. The control group included 30 age-matched healthy individuals. The IPF group had significantly higher proportions of men (90.9 vs 46.7%) and smokers (75.0 vs 16.7%) than the control group (All *p* < 0.01, Table 3). The average age was similar in two groups (IPF group: 62 ± 8 years, Control group: 61 ± 9 years) (Table 3).

**TABLE 3:** Patient general clinical data.

	IPF (*n* = 44)	Control (*n* = 30)
Men/women (ratio)	40/4 (10:1)^a^	14/16
Age (mean ± SD), year	62 ± 8	61 ± 9
Smoker, n (%)	33 (75.0)^a^	5 (16.7)
Disease duration (mean ± SD),week	28 ± 21	NA
CRP (mg/L)	7.5 ± 11.3	NA
ESR (mm/h)	32 ± 23	NA
PaO_2_ (mmHg)	80.5 ± 15.2	NA
PaCO_2_ (mmHg)	39.0 ± 3.8	NA
SaO_2_ (%)	95.1 ± 5.1	NA
FVC (L)	2.15 ± 0.90	NA
FVC, % pred	76.5 ± 28.2	NA
DLCO, % pred	58.2 ± 26.6	NA
Chest HRCT score	17.5 ± 7.6	NA
Daytime cough symptom score	1.26 ± 0.45	NA
Nighttime cough symptom score	0.65 ± 0.57	NA
Total cough symptom score	1.91 ± 0.90	NA

Control: healthy individuals. IPF = idiopathic pulmonary fibrosis, CRP = c-reactive protein, ESR = erythrocyte sedimentation rate, PaO_2_ = partial pressure of oxygen, PaCO_2_ = partial pressure of carbon dioxide, SaO_2_ = oxygen saturation, FVC = force vital capacity, FVC % pred = force vital capacity expressed as a percentage of predicted, D_L_CO % pred = carbon monoxide diffusing capacity of the lung expressed as a percentage of predicted, NA = not applicable, SD = standard deviation.

a Represent significant difference of the IPF group versus the control group, *p* < 0.01.

### Twenty-Four-hour Laryngopharyngeal pH Monitoring Results of the Three Groups

**FIGURE 2:**
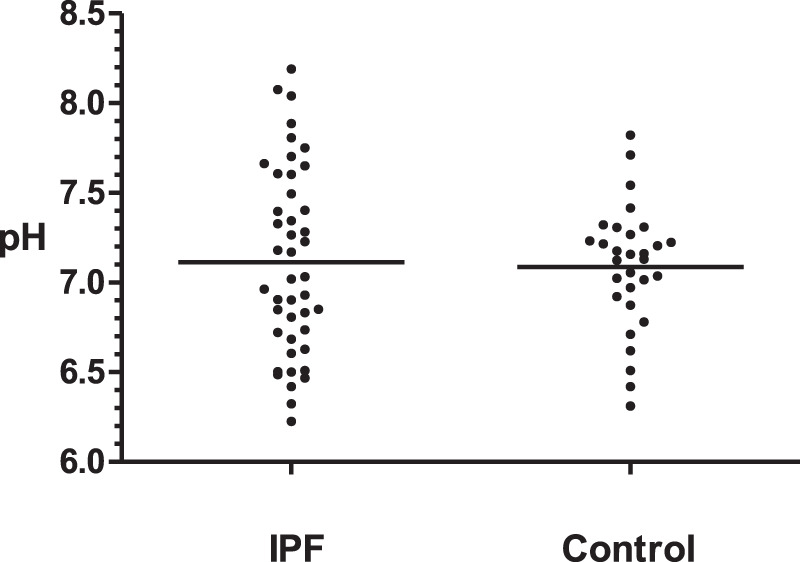
Overall mean laryngopharyngeal pH values of IPF and control groups Control: healthy individuals. IPF = idiopathic pulmonary fibrosis. The overall average laryngopharyngeal pH of two groups were similar (IPF: 7.11 vs Control 7.09, *p* > 0.05, Kruskal-Wallis test).

**FIGURE 3:**
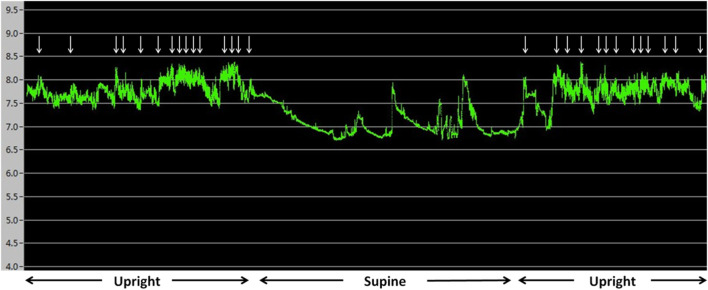
Results of 24 h pharyngeal pH monitoring of a patient with IPF The upright and supine periods can be identified easily by the pattern of the pH recording. pH was more than 7.5 for the majority of the monitoring time. ↓ represent cough events. Cough occurred when pH was >7.5.

The average laryngopharyngeal pH value for 24 h was similar in the IPF (7.11 ± 0.08) group and control group (7.09 ± 0.06) (Figure 2). The typical results of 24 h laryngopharyngeal pH monitoring of one patient with IPF are presented in Figure 3. We calculated the percentage duration of pH < 6.5, pH 6.5–7.5, and pH > 7.5 in the overall measurement time of every patient and allocated the 44 IPF patients and 30 healthy individuals into pH < 6.5, pH 6.5–7.5, and pH > 7.5 groups according to the highest percentage duration. When the entire pH measurement duration was used as the denominator to calculate the percentage duration, 11.4, 61.3, and 27.3% of the 44 IPF patients were in pH < 6.5, pH 6.5–7.5, and pH > 7.5 group, respectively. 3.3, 93.4, and 3.3% of the 30 healthy individuals were in each respective group. The proportion of pH > 7.5 group in IPF patients was higher than control group (*p* < 0.01); when the duration of upright position was used for the calculation, 6.8, 63.6, and 29.6% of the 44 IPF patients were in each respective group. 3.3, 93.4, and 3.3% of the 30 healthy individuals were in each respective group. The proportion of pH > 7.5 group in IPF patients was higher than control group (*p* < 0.01); when the duration of supine position was used, the proportions of 44 IPF patients in pH < 6.5, pH 6.5–7.5, and pH > 7.5 group were 27.3, 54.5, and 18.2%. The proportions of 30 healthy individuals in each respective group were 3.3, 90.0, and 6.7%. The proportion of pH < 6.5 group in IPF patients was higher than control group (Table 4). Overall, 7 patients showed abnormal Ryan index score at upright position (Ryan index score >9.41). None of the 44 patients showed abnormal Ryan index score at supine position (Table 5). Abnormal Ryan index score was defined as > 6.79.

**TABLE 4:** Proportions of patients in different pH groups.

	pH < 6.5, *n* (%)	pH 6.5–7.5, *n* (%)	pH > 7.5, *n* (%)
Entire pH monitoring duration			
Control, *n* = 30	1 (3.3)	28 (93.4)	1 (3.3)
IPF, *n* = 44	5 (11.4)	27 (61.3)	12 (27.3)^a^
At upright position			
Control, *n* = 30	1 (3.3)	28 (93.4)	1 (3.3)
IPF, *n* = 44	3 (6.8)	28 (63.6)	13 (29.6)^a^
At supine position			
Control, *n* = 30	1 (3.3)	27 (90.0)	2 (6.7)
IPF, *n* = 44	12 (27.3)^a^	24 (54.5)	8 (18.2)

Control: healthy individuals. IPF = idiopathic pulmonary fibrosis.

a Represent significant difference of the IPF group versus the control group, *p* < 0.01.

**TABLE 5:** Seven patients showing abnormal Ryan index score.

Patient no	Disease	Ryan index score	
		At the upright position	At the supine position^a^
1	IPF	65.33	2.17
2	IPF	11.5	2.17
3	IPF	59.82	2.17
4	IPF	11.91	2.17
5	IPF	32.15	2.17
6	IPF	17.5	2.17
7	IPF	23.91	2.17

IPF = idiopathic pulmonary fibrosis.

a When Ryan index score at the supine position was <6.79, the results from the calculation by the software were presented as 2.17.

### Correlation Between pH Value and Cough

No heart burn event occurred in the 44 IPF patients and 30 healthy individuals during the 24 h laryngopharyngeal pH monitoring. Comparison of the average pH value during coughing versus the overall average pH value showed that 4 IPF patients had significantly higher average pH at coughing than the overall average pH and one patient had significantly lower average pH at coughing (All *p* < 0.05, Table 6).

**TABLE 6:** Five patients showing significant changes in pH values at coughing.

Patient no	Disease	Overall average pH	Average pH at coughing	*p* value
1	IPF	7.6083 ± 0.2953	7.6992 ± 0.1093	0.015
2	IPF	7.1698 ± 0.4367	7.4450 ± 0.3122	0.021
3	IPF	6.7369 ± 0.3188	7.0700 ± 0.1950	0.009
4	IPF	7.3443 ± 0.2719	7.5490 ± 0.1906	0.008
5	IPF	6.8064 ± 0.4245	5.8867 ± 0.3667	0.049

IPF = idiopathic pulmonary fibrosis.

## Discussion

IPF is a progressive lung disease with an unknown etiology. The pathology of IPF is characterized by slowly progressive diffuse alveolar inflammation and/or alveolar structural disorders, which eventually damage alveolar structure and result in pulmonary fibrosis and honeycomb lung. IPF has a poor prognosis and the survival time of patients with IPF is approximately 3–5 years (Loomis-King et al., 2013). IPF ultimately lead to scars in lung tissues.

Previous studies have suggested that IPF may be highly associated with GERD [(Raghu et al., 2011), (Lee et al., 2011)]. Chronic inhalation of gaseous refluxate is a risk factor for airway and pulmonary inflammation and could induce or exacerbate IPF. Antacids, such as proton pump inhibitors and histamine H2-receptor antagonists, have been found to reduce the risk of GER-associated pulmonary damages (Raghu et al., 2006; Lee et al., 2011). Clinicians have routinely prescribed anti-acid drugs for patients with IPF (Raghu et al., 2011; Raghu et al., 2015). GER is caused by abnormal lower esophageal sphincter relaxation, which allows stomach contents to flow back to the esophagus. The majority of the stomach contents in GER are liquid and stay inside the esophagus. Patients with GER and LPR may experience gaseous reflux into the upper airway. LPR is caused by abnormal upper esophageal sphincter relaxation and is mainly gaseous reflux into the throat, nose, and ear. The gaseous refluxate can then enter the lower airway and alveoli during breathing. Thus, compared with GER, LPR appears more likely to adversely affect the lower airway and lung parenchyma and exacerbate IPF.

The etiology of GERD is associated with the dysfunction of gastric cardia and lower part of the esophagus. The clinical presentations of GERD are mainly digestive tract symptoms and occasional airway symptoms. The pathophysiology of GERD is characterized by acidic or basic gastric liquid and gaseous reflux into the esophagus and into the airway in severe cases. The etiology of LPR is associated with gastric empty dysfunction. LPR causes pathological changes in the airway. The clinical presentations of LPR are mainly airway symptoms but not digestive tract symptoms. The pathophysiology of LPR is characterized by acidic or basic gaseous reflux into the airway. Only 20% of patients with LPRD and GERD show low esophageal pH (the duration of pH < 4.0 is more than 4% of the total measurement time). Therefore, only monitoring esophageal pH could miss the diagnosis of LPRD in 80% of patients with LPRD (Ford, 2005; Yuksel and Vaezi, 2013). The 24 h laryngopharyngeal pH monitoring (DX-pH) has been used to diagnose LPRD (Sun et al., 2009; Wiener et al., 2009; Vailati et al., 2013). The diagnostic criteria for LPR based on DX-pH are the Ryan index at upright position >9.41 and/or at supine position >6.79.

In the current study, we monitored the laryngopharyngeal pH of 44 patients with IPF and 30 healthy individuals for 24 h and explored the association between IPF and LPR. The analysis of the overall 24 h laryngopharyngeal pH showed that 61.3, 27.3, and 11.4% of the 44 IPF patients had neutral, basic, and acidic laryngopharyngeal pH, respectively. 93.4, 3.3, and 3.3% of the 30 healthy individuals had neutral, basic, and acidic laryngopharyngeal pH. The proportion of pH > 7.5 group in IPF patients was higher than control group. The normal pH in the lower airway is 7.0–7.5. Thus, both basic reflux (pH > 7.5) and acidic reflux (pH < 6.5) appear to occur in patients with IPF. According to the diagnostic criteria for LPR (Ryan index at upright position >9.41), only 7 IPF patients met the criteria.

The normal pH in the lung is 7.0–7.5. pH > 7.5 may reduce enzyme activity in the lung tissues or even denature enzymes. Thus, basic reflux could affect lung function adversely, particularly for patients with IPF, who often have poor pulmonary elasticity because of lung fibrosis. Patients with IPF may have to inhale deeply to expand the alveoli because of the poor pulmonary elasticity. The deep inhalation may cause excessive negative pressure in the chest, which in turn may cause the basic contents including bile and pancreatic juice flow from the duodenal to the throat. The speed of this basic reflux may be too fast to allow the basic contents to be neutralized by gastric acid and to be cleared from the throat. The contents of basic refluxate and the adverse effects of basic refluxate on the laryngopharyngeal mucosa, the lower airway, and the lung need to be further investigated. Acidic reflux could activate the pepsin that has been deposited on the lower airway mucosa and alveoli and consequently result in nonspecific inflammation and trigger IPF.

The analysis of the pH at supine position showed that 54.5, 27.3, and 18.2% of the 44 IPF patients had neutral, acidic, and basic laryngopharyngeal pH, respectively. 90.0, 6.7, and 3.3% of the 30 healthy individuals had neutral, acidic, and basic laryngopharyngeal pH. The proportion of pH < 6.5 group in ILD patients was higher than control group. The supine position may actually facilitate gastric acid reflux into the throat. We found that using Ryan index only diagnosed 7 cases of LPR. For the entire pH monitoring duration and the duration at upright position, about 30% of the patients showed basic laryngopharyngeal pH, whereas for the duration at supine position, approximately 30% of the patients had acidic laryngopharyngeal pH. These findings indicate that the cut-off value of Ryan index for positive LPR (pH < 5.5 at upright position and/or pH < 5.0 at supine position) may not reflect LPR effectively in patients with IPF. In addition, we found that only five of the 44 patients showed significant difference between the average pH at coughing and the overall average pH. This suggests that cough appears more likely to be associated with IPF but not with LPR in patients with IPF. But it is not definitely clear whether the correlation between chronic cough and laryngopharyngeal pH changes is the causal effect or just correlation. We will further explore it in our future study. This study is an exploratory research. The sample size was relatively small. Idiopathic pulmonary fibrosis is a rare disease. Researches about laryngopharyngeal pH monitoring in patients with IPF were rarer. However, we still got some valuable findings in a limited number of study participants. Although the interpretation of the data is limited by small number of study participants, the current study was first to explore the possible association between LPR and IPF. In addition, we will investigate the composition of gaseous refluxate and study possible mechanism underlying the adverse effects of LPR on IPF initiation and development in our future study.

## Conclusion

We found that 50–60% of the patients had neutral laryngopharyngeal pH and did not need anti-acid treatments. Basic LPR may likely occur at upright position, whereas acidic LPR may probably occur at supine position. Patients with acidic LPR may use antacids (Park et al., 2005; Sato, 2006; Naik and Vaezi, 2013) and gastric pro-motility drugs (Glicksman et al., 2014). Patients with basic LPR may only need gastric pro-motility drugs. Ryan index score calculated based on acidic LPR may not reflect LPR accurately in patients with IPF.

## Abbreviations

CRP, c-reactive protein; CSS, cough symptom score; CTD-ILD, connective tissue disease-related interstitial lung disease; D_L_CO % pred, carbon monoxide diffusing capacity of the lung expressed as a percentage of predicted; EER, extraesophageal reflux; ESR, erythrocyte sedimentation rate; FVC % pred, force vital capacity expressed as a percentage of predicted; FVC, force vital capacity; GERD, gastroesophageal reflux disease; ILD, interstitial lung diseases; IPF, idiopathic pulmonary fibrosis; LPR, laryngopharyngeal reflux; PaCO_2_, partial pressure of carbon dioxide; PaO_2_, partial pressure of oxygen; SaO_2_, oxygen saturation.

## Data Availability

The raw data supporting the conclusions of this article will be made available by the authors, without undue reservation.
